# eIF2α phosphorylation bypasses premature senescence caused by oxidative stress and pro-oxidant antitumor therapies

**DOI:** 10.18632/aging.100620

**Published:** 2013-12-09

**Authors:** Kamindla Rajesh, Andreas I. Papadakis, Urszula Kazimierczak, Philippos Peidis, Shuo Wang, Gerardo Ferbeyre, Randal J. Kaufman, Antonis E. Koromilas

**Affiliations:** ^1^ Lady Davis Institute for Medical Research, McGill University, Sir Mortimer B. Davis-Jewish General Hospital, Montreal, Quebec H3T 1E2, Canada; ^2^ Department of Cancer Immunology, Chair of Medical Biotechnology, Poznan University of Medical Sciences, Poland; ^3^ Département de Biochimie, Université de Montréal; Montréal, Québec H3C 3J7, Canada; ^4^ Center for Neuroscience, Aging and Stem Cell Research, Sanford Burnham Medical Research Institute, La Jolla, CA 92037, USA; ^5^ Department of Oncology, Faculty of Medicine, McGill University, Montreal, Quebec H2W 1S6, Canada

**Keywords:** eIF2, protein phosphorylation, mRNA translation, reactive oxygen species, DNA damage, cellular senescence, doxorubicin

## Abstract

Eukaryotic cells respond to various forms of stress by blocking mRNA translation initiation via the phosphorylation of the alpha (α) subunit of eIF2 at serine 51 (S51) (eIFαP). An important role of eIF2αP is the regulation of redox homeostasis and adaptation of cells to oxidative stress. Herein, we demonstrate that eIF2αP guards cells from intracellular reactive oxygen species (ROS) via the inhibition of senescence. Specifically, genetic inactivation of either eIF2αP or eIF2α kinase PERK in primary mouse or human fibroblasts leads to proliferative defects associated with increased DNA damage, G_2_/M accumulation and induction of premature senescence. Impaired proliferation of either PERK or eIF2αP-deficient primary cells is caused by increased ROS and restored by anti-oxidant treatment. Contrary to primary cells, immortalized mouse fibroblasts or human tumor cells become tolerant to elevated intracellular ROS levels caused by impaired eIF2αP. However, eIF2αP-deficient human tumor cells are highly susceptible to extrinsic ROS generated by the pro-oxidant drug doxorubicin by undergoing premature senescence. Our work demonstrates that eIF2αP determines cell destiny through its capacity to control senescence in response to oxidative stress. Also, inhibition of eIF2αP may be a suitable means to increase the anti-tumor effects of pro-oxidant drugs through the induction of senescence.

## INTRODUCTION

Metazoans respond to various forms of stress by phosphorylating the α subunit of the eukaryotic initiation factor 2 (eIF2) at the serine 51 (herein referred to as eIF2αP), a modification that causes a general inhibition of protein synthesis [1,2]. In mammalian cells, eIF2αP is mediated by a family of four kinases each of which responds to distinct stimuli [1]. The family consists of the heme-regulated inhibitor (HRI), the general control non-derepressible-2 (GCN2), the endoplasmic reticulum (ER)-resident protein kinase PERK and the RNA-dependent protein kinase PKR [1,2]. These enzymes exhibit significant sequence similarities, particularly in the kinase domain (KD), which explains their specificity towards eIF2α [1,2]. Despite the general shutdown of protein synthesis, certain mRNAs like those encoding the activating transcription factor 4 (ATF4) in mammals and the general control non-derepressible-4 (GCN4) in yeast are efficiently translated under conditions of increased eIF2αP. This is because the 5′ untranslated regions (5′ UTRs) of these mRNAs consist of upstream open reading frames (uORFs), which impede translation of the downstream ORFs [1,2]. Increased eIF2αP decreases the formation of the eIF2-GTP-Met-tRNAi ternary complex, an event that bypasses the inhibitory effects of the uORFs leading to efficient re-initiation at the downstream ORFs [1,2].

PERK is implicated in the unfolded protein response (UPR), which is induced when the physiological environment of the ER is altered and the folding and maturation of secretory-pathway proteins is disrupted [3]. Activation of the PERK-eIF2αP branch of UPR switches off general protein synthesis but at the same time increases ATF4 synthesis, which in turn induces the transcription of genes that facilitate adaptation of cells to stress. An important property of PERK and eIF2αP is the regulation of redox homeostasis and adaptation of cells to oxidative stress caused by reactive oxygen species (ROS) formation in the intracellular environment. This property is mainly mediated by the increased synthesis and transcriptional activity of ATF4, which results in the expression of anti-oxidant genes [4,5]. Similar anti-oxidant mechanisms have been described in yeast in which increased eIF2αP induces mRNA translation of the transcriptional factor GCN4, which mediates expression of genes involved in adaptation to amino acid starvation and alleviation of oxidative damage from nutrient deprivation [6,7]. Increased ATF4 levels also contribute to restoration of protein synthesis following its inhibition by increased eIF2αP in response to stress [8,9]. However, if restoration of protein synthesis occurs before the recovery of protein folding capacity of the ER, increased intracellular ROS levels from protein misfolding utilizes ATF4 to orchestrate a pro-apoptotic program that selectively eliminates stressed cells [9]. Thus, induction of eIF2αP-ATF4 axis limits the deleterious effects of ROS by either increasing the anti-oxidant capacity of the cell or eliminating it by apoptosis when restoration of redox homeostasis in the ER is not possible.

Cellular senescence is a fundamental mechanism of aging and age-related diseases including cancer [10]. The ability of cells to induce UPR declines with aging indicating that cellular senescence can compromise adaptation processes associated with increased ER stress [11]. Also, senescence is an inherent tumor suppressive mechanism adapted by cells against neoplastic transformation; in tumor cells senescence can be prematurely induced by treatments with drugs causing genotoxic and/or oxidative stress [12]. Although the mechanisms of redox regulation by PERK and eIF2αP have been described [4,5], the biological consequences of the anti-oxidant function of both proteins have not been fully explored. Herein, we show that a key characteristic of the anti-oxidant function of the PERK-eIF2αP arm is the inhibition of premature senescence. We also show that primary and tumor cells respond differently to the anti-oxidant effects of PERK and eIF2αP. Specifically, the PERK-eIF2αP arm determines the sensitivity of primary cells to intracellular levels of ROS; primary cells defective in either PERK or eIF2αP are subjected to inhibition of cell cycle progression and induction of senescence. On the other hand, immortalized or tumor cells are tolerant to increased levels of endogenous ROS caused by impaired eIF2αP. Nevertheless, eIF2αP-deficient tumor cells become increasingly susceptible to exogenous ROS leading to inhibition of proliferation via the induction of senescence. The therapeutic implications of our study are supported by the findings that human tumor cells deficient in eIF2αP are increasingly susceptible to pro-oxidant effects of the anti-tumor drug doxorubicin through the induction of senescence.

## RESULTS

### Inactivation of either eIF2αP or PERK results in the induction of premature senescence

**Figure 1:**
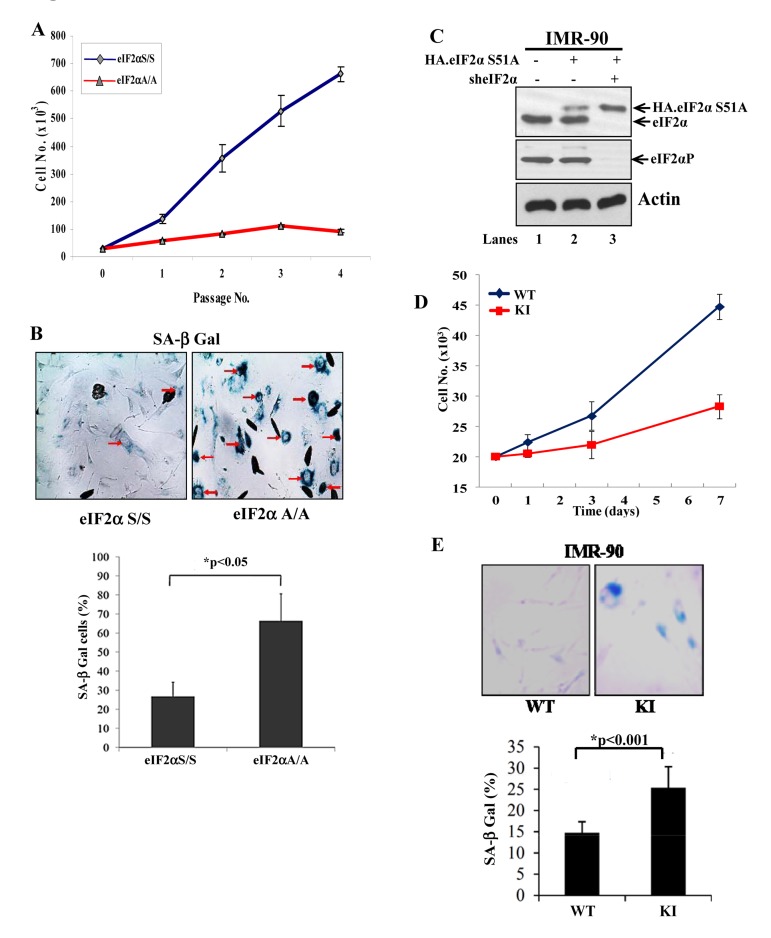
Loss of eIF2αP impairs proliferation and induces premature senescence in primary cells (**A**) Primary eIF2αS/S and eIF2αA/A MEFs were maintained in culture for the indicated passages. Proliferation was measured by cell counting. Values represent an average taken from two independent experiments performed in triplicates. (**B**) Senescence in eIF2αS/S and eIF2αA/A MEFs was monitored by SA β-Gal staining. The ratio of the percentage of SA-β-gal positive cells obtained from two independent experiments is as indicated in the histogram. Senescent cells are indicated by arrows. (**C**) Primary IMR90 human fibroblasts were engineered to express an HA-tagged eIF2αS51A (lanes 2 and 3) under conditions in which endogenous eIF2α was down regulated by shRNA expression (lane 3; KI cells). Protein extracts (50 μg) were subjected to immunoblot analysis for the indicated proteins. Note that the slow-migrating band detected by the anti-eIF2α antibody corresponds to HA-eIF2αS51A (lanes 2, 3) and that endogenous eIF2α was substantially down regulated by shRNA expression (lane 3). Control IMR90 cells (WT) in lane 1 represent cells which were infected with insert-less retroviruses and lentiviruses. (**D**) Proliferation of IMR90 WT and KI cells was measured by cell counting. Values represent an average of two independent experiments performed in triplicates. (**E**) Cells were stained for SA β-Gal and the average percentage values of positive cells from three independent experiments are indicated. Senescent cells are in blue color.

We were interested to examine the effects of impaired eIF2αP on the proliferation of primary mouse and human cells. We observed that primary mouse embryonic fibroblasts (MEFs) bearing a substitution of serine 51 to alanine (S51A) in both eIF2α alleles (herein referred to as eIF2αA/A cells) exhibited a substantially lower proliferation capacity than the isogenic primary MEFs with a wild-type eIF2α allele (i.e. eIF2α S/S cells) (Fig. 1A). The eIF2αA/A MEFs ceased proliferating in early passages and displayed an enlarged, flattened morphology characteristic of senescent cells as indicated by increased staining for senescence associated β-galactosidase (SA-βGal) (Fig.1B). Similar observations were made with human diploid IMR-90 fibroblasts engineered to express an HA-tagged form of eIF2αS51A under conditions of which endogenous eIF2α was down regulated by shRNA [herein referred to as knock-in (KI) cells; Figs. 1C, D]. We saw that IMR-90 KI cells proliferated slower and exhibited increased staining for SA-β-Gal compared to control IMR-90 cells with wild-type (WT) eIF2α (Fig. 1E). These data suggested that impaired eIF2αP inhibits proliferation and induces premature senescence in mouse and human primary fibroblasts.

**Figure 2:**
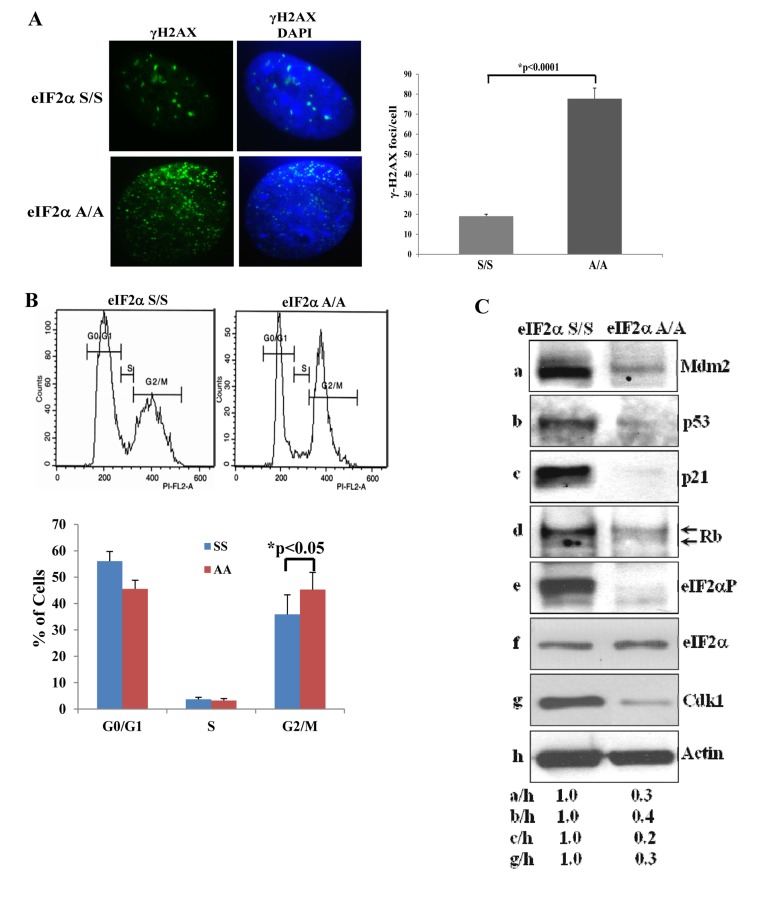
Impaired eIF2α phosphorylation is associated with increased DNA Damage and G/M arrest (**A**) eIF2αS/S and eIF2αA/A MEFs from passage 3 were stained for γ-H2AX and analyzed by fluorescence microscopy. DAPI staining was used to visualize nuclei. Histograms show the average number of γ-H2AX foci per cell (n=100). (**B**) eIF2αS/S and eIF2αA/A MEFs from passage 3 were subjected to cell cycle analysis by FACS. Histograms indicate the percentages of cells in G_0_/G_1_, S and G_2_/M from three independent experiments. (**C**) Protein extracts (50 μg) from eIF2αS/S and eIF2αA/A MEFs from passage 3 were immunoblotted for the indicated proteins.

To better address the anti-proliferative properties of eIF2αP-deficient cells, we focused on mouse cells and examined the levels of DNA damage, which is a hallmark of senescence. We looked at the phosphorylation of H2AX at serine 139 [herein referred to as gamma (γ) H2AX], which is an established marker of the DNA damage response. We found that eIF2αA/A MEFs displayed a substantially increased level of γ-H2AX staining compared to eIF2αS/S MEFs (Fig. 2A). We also examined the cell cycle distribution of MEFs and found that eIF2αA/A MEFs displayed a decrease in G_0_/G_1_ and increase in G_2_/M compared to eIF2αS/S MEFs (Fig. 2B). Increased G_2_/M was an unexpected result because mouse fibroblasts undergoing senescence usually arrest in G_0_/G_1_ due to activation of p53 and/or Rb [13]. Consistent with the down regulation of cells in G_0_/G_1_, we found that p53 together with its transcriptional targets p21 and Mdm2 were substantially decreased in eIF2αA/A MEFs compared to eIF2αS/S MEFs (Fig. 2C). We also observed that the hyper- and hypo-phosphorylated forms of Rb, which can be distinguished by their different migration in polyacrylamide gels, were decreased in eIF2αA/A MEFs compared to eIF2αS/S MEFs (Fig. 2C). Furthermore, we noticed that eIF2αA/A MEFs contained lower levels of the cyclin-dependent kinase 1 (Cdk1), an event that could account for the increased G_2_/M of the cells (Figs. 2B,C).

**Figure 3:**
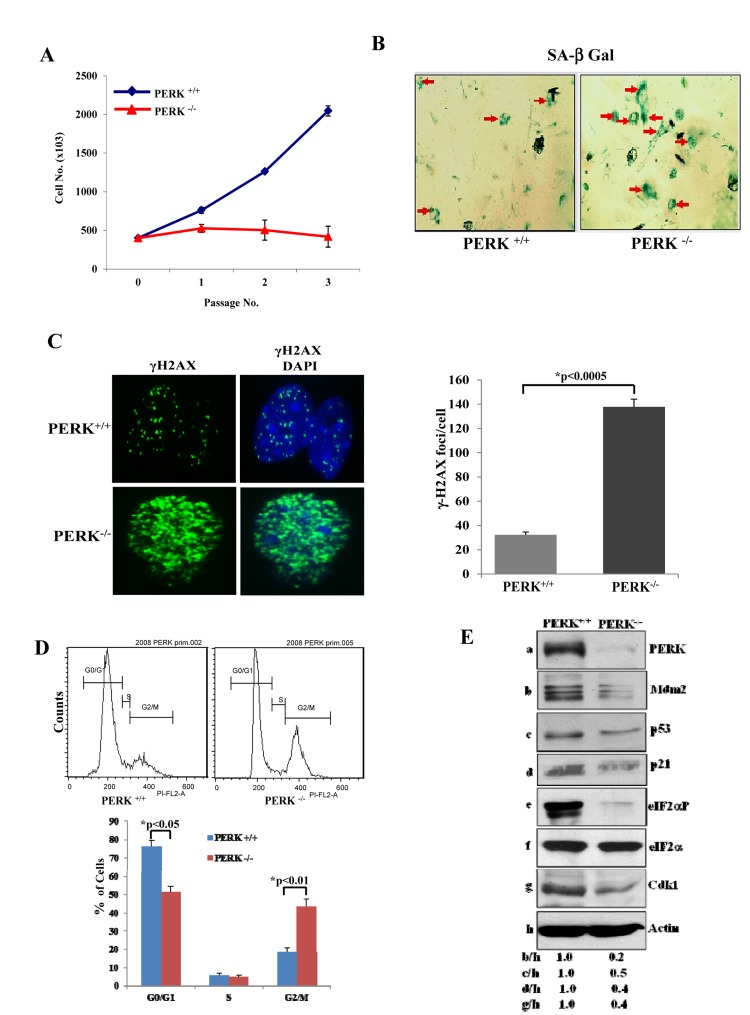
PERK deficiency impairs proliferation and induces premature senescence (**A**) Primary PERK^+/+^ and PERK^−/−^ MEFs were maintained in culture for the indicated passages and their proliferation was assessed by cell counting. The data represent an average taken from two independent experiments performed in triplicates. (**B**) Induction of senescence was evaluated by SA β-Gal staining from cells in passage 3. Senescent cells are indicated by arrows. (**C**) DNA damage was assessed by γ-H2AX staining and fluorescence microscopy of cells in passage 3. Nuclei were visualized by DAPI staining. Histograms represent the average number of γ-H2AX foci per cell (n=100). (**D**) PERK^+/+^ and PERK^−/−^ MEFs from passage 3 were subjected to PI staining and FACS analysis to determine cell cycle progression. Histograms show the percentages of cells in G_0_/G_1_, S and G_2_/M from three independent experiments. (**E**) Protein extracts (50 μg) from MEFs maintained in passage 3 were immunoblotted for the indicated proteins.

PERK plays a major role in the adaptation of cells to stress via the induction of eIF2αP. Consistent with this notion, we observed that genetic loss of PERK significantly reduced the proliferative capacity of primary MEFs (i.e. PERK^−/−^ MEFs) compared to isogenic primary PERK^+/+^ MEFs (Fig. 3A). Inhibition of PERK^−/−^ cell proliferation was accompanied by an induction of senescence as indicated by increased staining of cells for SA-β-Gal (Fig. 3B). Moreover, PERK^−/−^MEFs displayed increased levels of DNA damage and noticeable differences in cell cycle distribution as indicated by a decrease in G_0_/G_1_ and increase in G_2_/M compared to isogenic PERK^+/+^ MEFs (Figs. 3C,D). Furthermore, Cdk1 as well as p53 together with Mdm2 and p21 were down regulated in PERK^−/−^ MEFs compared to PERK^+/+^ MEFs (Fig. 3E). Moreover, eIF2αP was substantially decreased in PERK^−/−^ MEFs compared to PERK^+/+^ MEFs (Fig. 3E). The significant similarities between PERK^−/−^ and eIF2αA/A MEFs as well as the substantial loss of eIF2αP in PERK^−/−^ MEFs supported the interpretation that PERK plays a major role in the inhibition of premature senescence mediated by eIF2αP.

### Induction of premature senescence by impaired eIF2αP is caused by increased intracellular ROS levels

**Figure 4:**
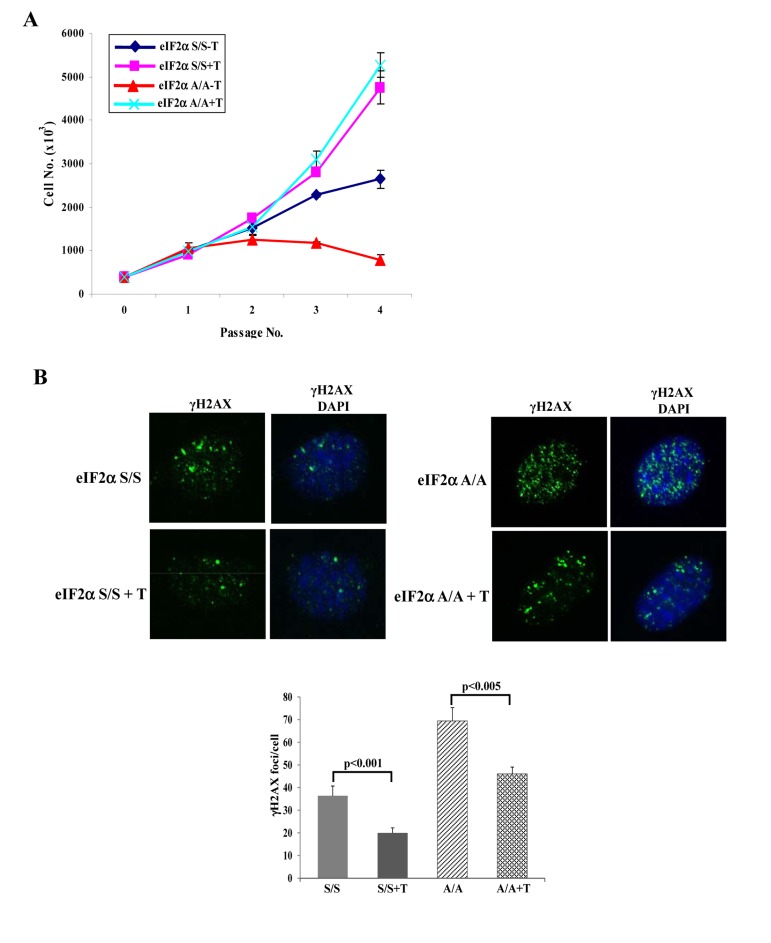
Anti-oxidant treatment restores proliferation and alleviates DNA damage in eIF2αA/A cells (**A**) Primary eIF2αS/S and eIF2αA/A MEFs were maintained in the absence or presence of 200 μM Trolox (T). Cell proliferation for the indicated passages was assessed by cell counting. Data represent an average taken from two independent experiments performed in triplicates. (**B**) Primary eIF2αS/S and eIF2αA/A MEFs MEFs in the absence or presence of 200 μM Trolox in passage 3 were subjected to γ-H2AX staining and fluorescence microscopy. Nuclei were visualized by DAPI staining. Histogram represents the average number of γ-H2AX foci per cell (n=100).

Cells deficient in eIF2αP display increased rates of general protein synthesis, which are associated with ER overload and accumulation of improperly folded proteins [14]. Protein misfolding in the ER can lead to increased ROS production [15], a process that is significantly facilitated by defects in either eIF2αP or PERK [4,5,14]. Consistent with these findings, we observed that eIF2αA/A or PERK^−/−^ MEFs contained increased ROS levels compared to their isogenic control MEFs (Suppl. Fig. 1). Since ROS significantly contributes to DNA damage and induction of senescence [16], we hypothesized that ROS was a cause of senescence in MEFs deficient in either PERK or eIF2αP. We addressed this matter in primary eIF2αA/A MEFs by assessing their proliferative capacity in the absence or presence of Trolox, a derivative of vitamin E with anti-oxidant function [17]. We observed that Trolox substantially improved the proliferation rates of eIF2αA/A MEFs and restored them to levels that were nearly identical to eIF2αS/S MEFs (Fig. 4A). Also, treatment with Trolox significantly decreased the DNA damage response in eIF2αA/A MEFs compared to eIF2αS/S MEFs confirming its anti-oxidant function and indicating that increased ROS production contributes to increased DNA damage (Fig. 4B).

### Immortalized and tumor cells deficient in eIF2αP are tolerant to increased levels of intracellular ROS

**Figure 5:**
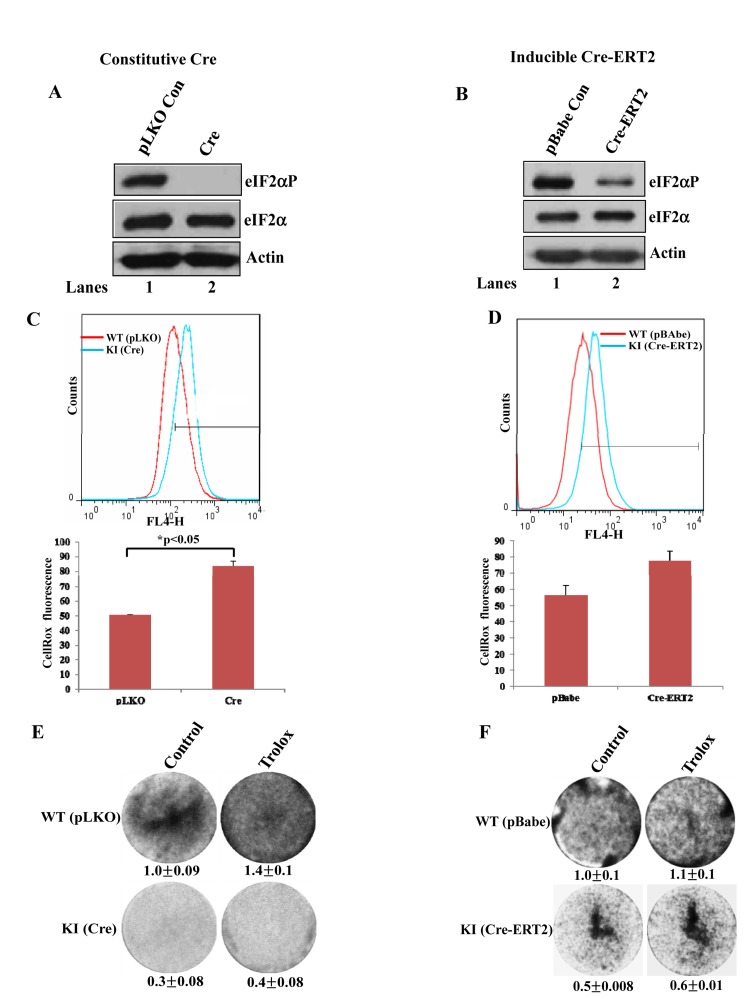
Increased ROS levels in eIF2αP-deficient immortalized cells (**A, B**) Primary fTg/eIF2αA/A MEFs were subjected to immortalization by the expression of SV40 large T Ag followed by lentivirus-mediated expression of Cre (A) or retrovirus-mediated expression of Cre-ERT2 (B). Cre-ERT2 cells were treated with 1μM tamoxifen for 72 hours (B, lane 2). As control, cells were infected with insert-less retroviruses (A,B, lane 1) in the presence of 1 μM tamoxifen (B, lane 1). Protein extracts (50 μg) from proliferating cells were used for immunoblot analysis for the indicated proteins. (**C,D**) ROS levels were measured in immortalized fTg/eIF2αA/A MEFs expressing either Cre-ERT2 (C) or constitutive Cre (D) by Cell-RoxTM Deep Red staining and FACS analysis. Histograms represent the average ROS levels measured by CellRox fluorescence in three independent experiments. (**E,F**) Colony formation assays of untreated as well as Trolox-treated immortalized fTg/eIF2αA/A MEFs expressing a constitutive Cre (E) or a tamoxifen inducible Cre-ERT2 (F). Cells were visualized by crystal violet staining. Values represent ratios of optical density (OD) in arbitrary units.

Immortalized and tumorigenic cell lines are generally thought to be refractory to the anti-proliferative effects of intracellular ROS [16]. As such, we wished to examine whether impaired eIF2αP had an effect on intrinsic ROS levels and proliferation of immortalized cells. We employed primary MEFs from mice engineered to express a floxed/floxed WT eIF2α transgene in eIF2αA/A background (herein referred to as fTg/eIF2αA/A cells) [4]. Expression of Cre recombinase in fTg/eIF2αA/A MEFs causes deletion of eIF2α WT transgene leading to expression of the non-phosphorylatable eIF2αS51 under the control of the endogenous eIF2α gene promoter [4]. Primary fTg/eIF2αA/A MEFs were first immortalized by the expression of SV40 large T antigen followed by either the constitutive expression of Cre (Fig. 5A) or the expression of a Cre chimera protein with the hormone binding domain of the estrogen receptor (Cre-ERT2) (Fig. 5B). We noticed that downregulation of eIF2αP by both approaches resulted in increased ROS production, which was proportional to the degree of impaired eIF2αP (Figs. 5C, D). Also, increased ROS production was associated with an inhibition of cell proliferation compared to control cells (Figs. 5E,F). However, the differences in proliferation between eIF2αP-proficient and deficient MEFs were maintained after Trolox treatment indicating that the diminished proliferative capacity of eIF2αP-deficient cells was not caused by the upregulation of intracellular ROS (Figs. 5E,F).

**Figure 6:**
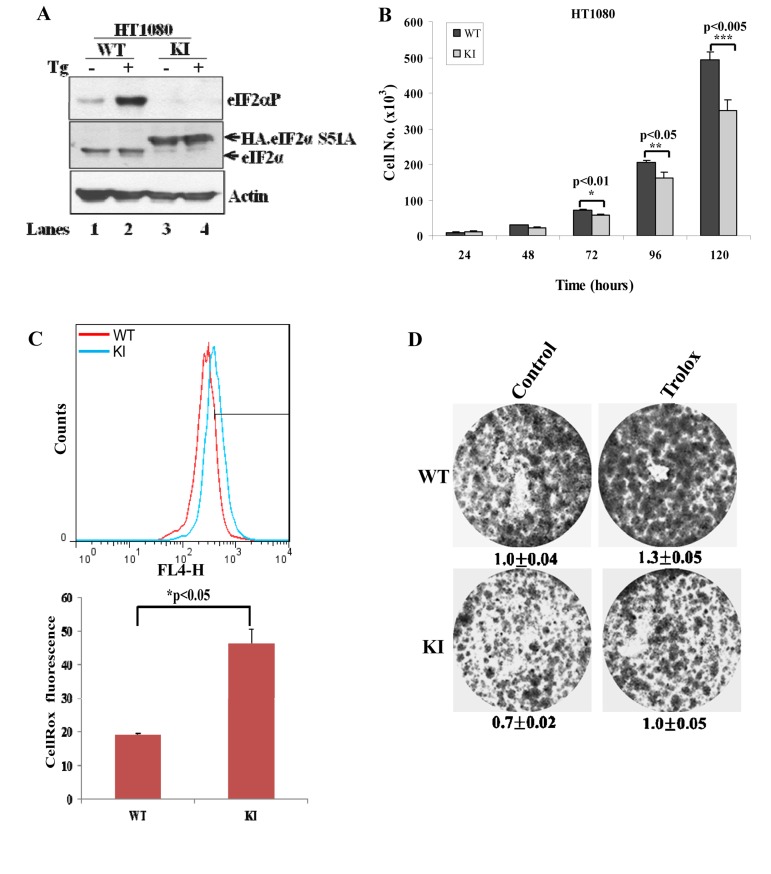
Inactivation of eIF2αP in human tumor cells decreases cell proliferation and increases ROS levels (**A**) Cell extracts (50 μg of protein) from wild-type (WT) and knock-in (KI) HT1080 cells treated with 1μM thapsigargin (TG) for 2 hours were subjected to immunoblot analysis for the indicated proteins. Note the slower migration of the HA-eIF2αS51A in KI cells (lanes 3,4) compared to endogenous eIF2α in WT cells (lanes 1,2). (**B**) Cell proliferation of HT1080 WT and KI cells was assessed by cell counting and was plotted over time. The error bars indicate the standard deviation. (**C**) ROS levels in HT1080 WT and KI cells was assessed by Cell-RoxTM Deep Red staining and FACS analysis. Histograms represent the average ROS levels measured by CellRox fluorescence from three independent experiments. (**D**) Colony formation assays of HT1080 WT and KI cells in the absence or presence of 200 μM Trolox. Cells were stained with crystal violet. Values represent ratios of optical density (OD) in arbitrary units.

We made similar observations in tumor cell lines, that is, in human fibrosarcoma HT1080 cells and lung adeno-carcinoma A549 cells, both of which were engineered to be deficient in eIF2αP (i.e. KI cells) (Fig. 6A; Suppl. Fig. 2A). Specifically, we observed that KI tumor cells displayed reduced proliferation and increased ROS production compared to WT cells (Figs. 6B,C; Suppl Fig. 2B). However, treatment with Trolox increased the proliferation capacity of both WT and KI cells but did not restore the proliferation of KI cells to the same levels as of WT tumor cells (Fig.6D; Suppl. Fig. 2C). This finding suggested that eIF2αP-deficient tumor cells were equally sensitive to the anti-proliferative effects of intracellular ROS as eIF2αP-proficient tumor cells.

### Tumor cells deficient in eIF2αP are increasingly susceptible to ROS-mediated induction of senescence in response to doxorubicin

**Figure 7:**
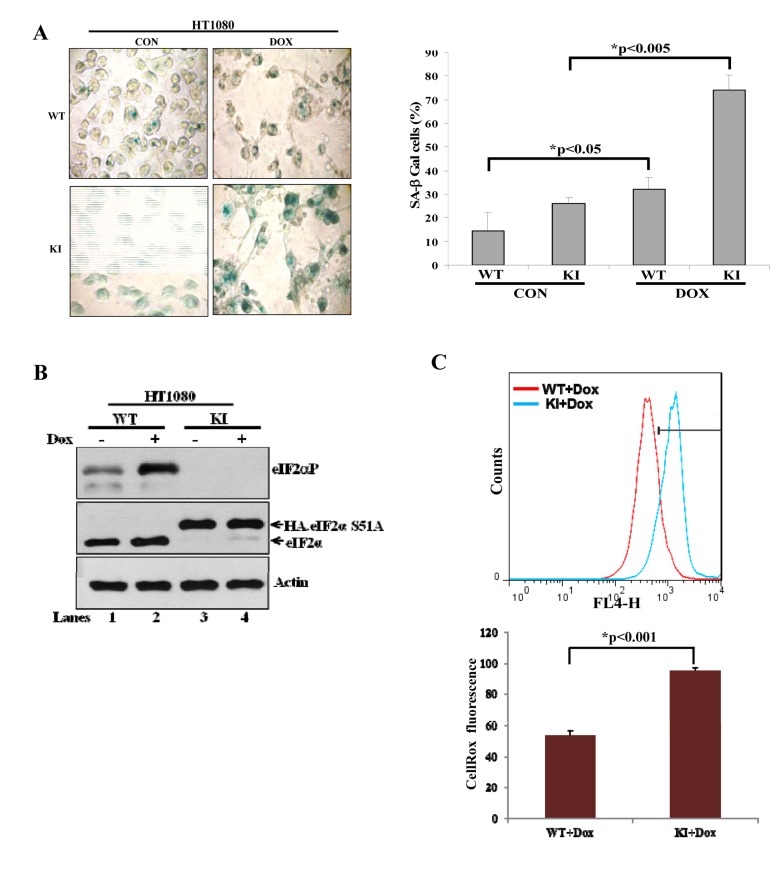
Deficiency of eIF2αP sensitizes human tumor cells to pro-oxidant effects of doxorubicin (**A**) HT1080 WT and KI cells were treated with 30 nM doxorubicin (Dox) for 36 hours. Untreated as well as doxorubicin treated cells were subjected to staining for SA-β-Gal. Average values of the percentages of SA-β-Gal positive cells from three independent experiments are shown in the histograms. (**B**) Protein extracts (50 μg) from untreated or doxorubicin (30 nM) treated WT and KI cells were immunoblotted for indicated proteins. HA-eIF2αS51A in KI cells (lanes 3,4) migrates slower compared to endogenous eIF2α in WT cells (lanes 1,2). (**C**) Endogenous ROS levels in HT1080 cells WT and KI treated with 30 nM doxorubicin were measured by CellRox fluorescence. Histograms represent the average ROS levels from three independent experiments.

**Figure 8:**
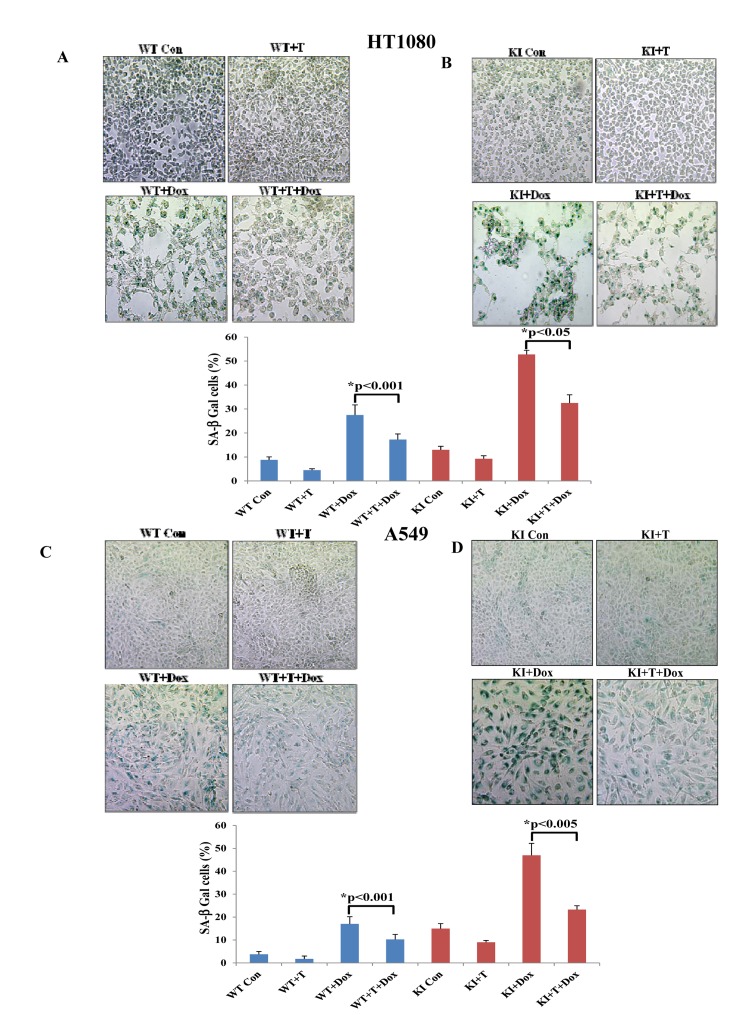
ROS contributes to doxorubicin-mediated induction of senescence in tumor cells HT1080 WT and KI cells (**A,B**) as well as A549 WT and KI cells (**C,D**) were treated with 30 nM doxorubicin (Dox) in the absence (Con) or presence of 200 μM Trolox (T) for 36 hours. Cells were stained for SA β-Gal; histograms represent the average percentage values of SA β-Gal positive cells from three independent experiments. Senescent cells are shown in blue color.

Tumor cells can tolerate ROS up to a certain level above which they become sensitive to the anti-proliferative effects of ROS [18]. This has been considered an important property of tumor cells, which can be exploited in anti-tumor therapies with pro-oxidant drugs [18]. Thus, we were interested to examine whether eIF2αP could determine the sensitivity of tumor cells to excessive oxidative stress. Doxorubicin is a widely prescribed anti-tumor drug that induces ROS synthesis and acts as an inducer of senescence when applied in sub-lethal amounts [19]. We observed that treatment of HT1080 with 30 nM doxorubicin did not cause cell death but rather resulted in deregulation of cell cycle as indicated by an increased accumulation of tumor cells in G_2_/M, which was more noticeable for KI than WT tumor cells (Suppl Fig. 3). We also observed that treatment with the sub-lethal concentration of doxorubicin resulted in the induction of SA-β-Gal staining in HT1080 cells, which was more evident for KI than in WT tumor cells (Fig. 7A). We further noticed that doxorubicin treatment increased eIF2αP in WT tumor cells but not in KI tumor cells (Fig. 7B) and resulted in a higher amount of ROS synthesis in KI than in WT tumor cells (Fig. 7C; compare it with Fig. 6C). This data indicated that induction of senescence by doxorubicin was enhanced by the loss of eIF2αP and coincided with increased ROS production in eIF2αP-deficient cells compared to eIF2αP-proficient cells. To address the role of ROS in the induction of senescence, HT1080 and A549 cells were subjected to doxorubicin treatment in the absence or presence of Trolox. We found that incubation with the anti-oxidant decreased the background levels of SA-β-gal in both tumor cell types and resulted in a substantial reduction of SA-β-Gal staining in doxorubicin treated tumor cells (Fig. 8). Interestingly, Trolox treatment caused a higher reduction in SA-β-Gal staining in doxorubicin-treated KI tumor cells than in WT tumor cells indicating that increased ROS synthesis in KI cells contributes to a higher induction of senescence than in WT tumor cells (Fig. 8). Collectively, these data suggested that ROS production contributes to doxorubicin-mediated senescence of tumor cells, a process that is substantially enhanced by the loss of eIF2αP.

**Figure 9:**
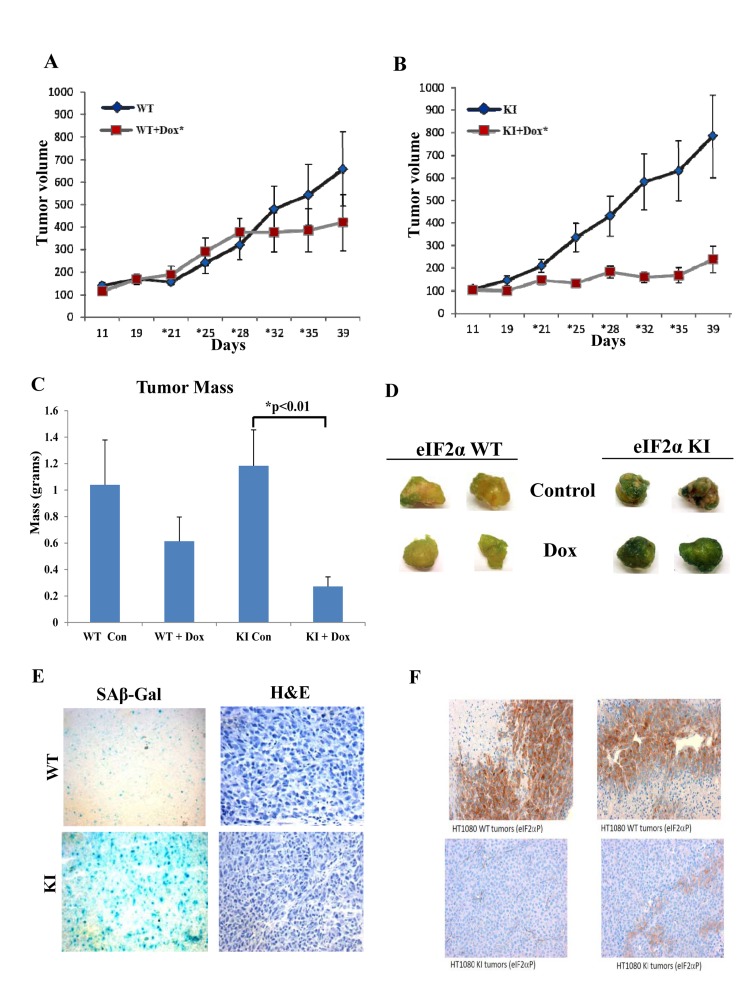
Deficient eIF2αP inhibits growth and promotes senescence of doxorubicin treated human tumors in mice (**A,B**) HT1080 WT and KI tumor cells were injected subcutaneously in the flanks of 10 female nude mice for each group. Each mouse received two subcutaneous injections (1×10^5^ cells per injection site) in the abdomen proximal to the rear limbs (n=2×5=10). After injection tumors were left to grow to a measurable size and half of mice (n=5) from each group were treated with placebo and the other half with 4 mg/kg doxorubicin. Tumor growth was monitored for 40 days. Asterisks indicate the time points of doxorubicin injections. (**C**) At the endpoint of the experiment, tumors were excised from the mice and the mass of each tumor was determined. Histograms represent the average mass of tumors. (**D**) Equal-sized pieces of tumors were cut from HT1080 WT and KI tumors and subjected to SA-β-Gal staining. (**E**) Tumor sections from doxorubicin treated WT and KI tumors were subjected to SA β-Gal and H&E staining. (**F**) The levels of eIF2αP in the WT and KI tumors was assessed by staining of tumor sections with phospho-specific antibodies against Ser51.

To better assess the biological significance of the findings, we examined the effects of doxorubicin on tumor growth in xenograft tumor assays in nude mice. We observed that doxorubicin treatment resulted in a substantial reduction of growth of HT1080 KI cells compared to control WT cells during the observation period of 40 days (Figs. 9A, B). When the tumor masses were measured at the end point of the experiment, it became evident that while tumor growth between WT and KI cells did not significantly differ, treatment with doxorubicin resulted in a significant reduction of KI tumor mass compared to WT tumor mass (Fig. 9C). When tumors were cut into pieces of equal size and subjected to SA-β Gal staining, we noticed that doxorubicin treated KI tumors displayed higher levels of senescence than WT tumors (Fig. 9D). Increased levels of senescence in doxorubicin treated KI tumors was also evident after staining of tumor sections for SA-β Gal (Fig. 9E) whereas staining for eIF2αP verified that the KI state was maintained during tumor growth in nude mice (Fig. 9F). These data indicated that loss of eIF2αP promotes the anti-tumor effects of doxorubicin *in vivo* via the induction of senescence.

## DISCUSSION

The anti-oxidant function of eIF2αP depends on its translational properties and requires efficient ATF4 synthesis, which in turn induces transcription of genes involved in the import of thiol-containing amino acids and glutathione biosynthesis as a means to counteract oxidative insults [5]. In mammalian cells, ATF4 has additional transcriptional roles by acting alone or in combination with other transcription factors to induce the expression of anti-oxidant genes like heme oxygenase-1 and sequestosome1/A170 [5]. In a pathway different from eIF2αP, PERK can phosphorylate nuclear factor (erythroid-derived 2)-like 2 (NFE2L2), also known as ***NRF2***, and promote its dissociation from Keap1 resulting in increased translocation of active NRF2 to the nucleus [20]. Also, attenuation of general protein synthesis caused by increased eIF2αP is another mechanism that contains intracellular ROS levels and contributes to adaptation of cells to oxidative stress. This is because client protein load in the ER decreases, which in turn prevents illegitimate disulfide bond formation in the under chaperoned ER. As such, cells are supplied with a sufficient amount of reducing equivalents to alleviate themselves from oxidative stress [5]. Due to close proximity of the ER to mitochondria, activation of the PERK and increased eIF2αP have also been proposed to be involved in the regulation of mitochondrial ROS production. Specifically, attenuation of protein synthesis prevents cells from ATP depletion, which takes place when the ER is overwhelmed with misfolded client proteins [21]. Depletion of ATP stimulates mitochondrial oxidative phosphorylation and ROS production, which can be further induced by increased efflux of Ca^2+^ from ER to cytosol as a result of ER stress [22]. In line with this notion, a previous study provided evidence that mitochondrial function contributes to ROS production in eIF2αP-deficient pancreatic β cells [4].

Increased intracellular ROS levels from the inactivation of either PERK or eIF2αP in primary fibroblasts induces premature senescence consistent with previous studies showing that ROS is a strong inducer of cellular senescence [16]. The proliferative defects from eIF2αP inactivation were associated with increased accumulation of cells in G_2_/M in primary cells as well as in tumor cells treated with the pro-oxidant drug doxorubicin. Given that rates of protein synthesis decrease during mitosis [23,24], increased G_2_/M in eIF2αP-deficient cells may also be translational in nature owing to the efficient production of several proteins that impede entry to and progression through mitosis. Also, proper mitosis requires the formation and activation of the Cdk1/cyclin B complex in a timely fashion [23,24]. We observed that Cdk1 levels decreased in PERK- as well as eIF2αP-deficient cells implying that the Cdk1/cyclinB complex might not be fully active in these cells. Increased G_2_/M accumulation may also be facilitated by the down regulation of p53 and Rb, which allows eIF2αP-deficient cells to bypass G_0_/G_1_ arrest caused by the activation of the tumor suppressor proteins in response to DNA damage. Down regulation of p53 in primary MEFs may further contribute to ROS production and induction of senescence based on previous data showing that p53 can exert an anti-oxidant function via the transcriptional induction of sestrin 1 and 2 [25]. How p53 and Rb are down regulated by the loss of eIF2αP is not immediately clear. Down regulation of p53 and Cdk1 could be translational in nature given that increased eIF2αP can promote translation of specific cellular mRNAs via internal ribosome site entry (IRES)-mediated mechanisms [26]. Consistent with this notion, translation of both Cdk1 and p53 mRNAs is IRES-dependent [27-29] whereas p53 mRNA is efficiently translated under conditions of increased eIF2αP in spite of the general inhibition of protein synthesis [30]. Similar to eIF2αP deficiency, a previous study showed that ATF4-deficient primary MEFs are prone to premature senescence, which renders them refractory to oncogene-mediated transformation [31]. However, induction of senescence in ATF4-deficient MEFs required the activation of p53 and Rb [31]. Thus, although eIF2αP and ATF4 act together to elicit anti-oxidant functions, downstream pathways mediated by each protein can be diverse. This notion is further supported by recent findings showing that eIF2αP and ATF4 have different roles in the recovery of protein synthesis in response to ER stress [8,9].

We provide evidence that eIF2αP-deficient primary cells are sensitive to intrinsic ROS production and induction of senescence whereas immortalized mouse and human tumor cells deficient in eIF2αP are adapted to increased levels of intracellular ROS. It is of interest that inactivation of eIF2αP decreased the proliferation of immortalized mouse and human tumor cells independent of the increased intracellular ROS. The decreased proliferation capacity of eIF2αP-deficient cells can be explained by previous findings showing that loss of eIF2αP results in UPR upregulation owing to increased rates of protein synthesis and accumulation of misfolded proteins in the ER [14]. It has been thought that tumor cells have evolved mechanisms to protect themselves from intrinsic oxidative stress by rearranging the anti-oxidant functions and upregulating pro-survival pathways [32]. Changes in the redox status of tumor cells are often caused by increased metabolic activity required to maintain a high rate of proliferation [33]. In this regard, eIF2αP plays a role in the flux of amino acids as well as glycolytic intermediates required to support cell proliferation [5,9]. Therefore, the possibility remains that decreased proliferation of eIF2αP-deficient tumor cells is caused by metabolic alterations that impact on proliferation. Our data show that eIF2αP in tumor cells can act as a molecular sensor of ROS and determine cell fate when ROS levels exceed a certain threshold.

The prevailing idea about chemotherapy is to induce apoptosis in cancer cells, but it was also shown that cancer cells treated with sub-lethal doses of the drugs and/or radiation enter into senescence [12,34]. Because senescent cells are cleared efficiently by innate immune system leading to tumor regression [35], drug induced senescence is considered as a powerful therapeutic approach to treat cancers [12,34]. Herein, we show that tumor cells deficient in eIF2αP produce higher levels of ROS than tumor cells proficient in eIF2αP in response to doxorubicin resulting in the induction of senescence and inhibition of tumor growth *in vitro* and *in vivo*. In line with our findings, recent studies provided strong evidence that increased eIF2αP protects tumors from increased ROS production during cyclic hypoxia and contributes to their survival in response to irradiation therapy and/or chemotherapy [36]. Collectively, these data raise the interesting hypothesis that inhibition of eIF2αP may be a suitable means to increase the efficacy of anti-tumor therapies that promote oxidative stress. Interestingly, recent studies revealed a different role of eIF2αP in anti-tumor therapies that elicit immunogenic responses. Specifically, it has been shown that increased eIF2αP by DNA damaging agents contributes to the translocation of calreticulin (CRT) to the surface of the plasma membrane, which acts as a signal to immune cells for tumor clearance [37]. Because the tumorigenicity of human cancer cells was tested in immunodeficient mice, our study cannot address the immunesurveillance component of eIF2αP in response to doxorubicin. Our work examines the cell-autonomous function of eIF2αP, which is mediated by its ability to promote the survival and maintain the proliferation of tumor cells exposed to the oxidative drug. Considering that the immunogenic properties of CRT delay but do not abolish tumor formation [38], it remains possible that the cell-autonomous and pro-survival properties of eIF2αP are highly relevant for those tumors that escape from immune surveillance and develop resistance to immunogenic therapies. This interpretation is consistent with our previous work showing that eIF2αP is important for the survival of tumor cells exposed to pharmacological inhibitors of PI3K-Akt or Bcr Abl signaling [39,40] as well as with recently published work showing that eIF2αP promotes survival of a subset of hypoxic tumors that become resistant to radiation therapy [36]. Thus, a better understanding of the role of eIF2αP in defining the balance between immunogenic and non-immunogenic anti-tumor therapies will be important to design and implement therapeutic approaches that target eIF2αP as a means to combat cancer [41].

## EXPERIMENTAL PROCEDURES

### Cell culture and treatments

Primary mouse embryonic fibroblasts (MEFs) were derived from PERK^+/−^ or eIF2αS/A mice as described [4,14,42]. MEFs were maintained in Dulbecco modified Eagle medium (DMEM; Wisent) supplemented with 10% fetal bovine serum (FBS; Gibco), 1x of essential and non-essential amino acids (Gibco) and antibiotics (100 U/ml of penicillin-streptomycin; Gibco). IMR-90, HT1080 and A549 cells were cultured in DMEM supplemented with 10% FBS and antibiotics. Immortalization of primary MEFs was performed by infection of pBABE-retroviruses bearing SV40 large T antigen (Addgene) and selection in 2.5 μg/ml puromycin (Sigma). Immortalized fTg/AA MEFs were transduced with either the pLKO.1-Cre lentiviral vector (Lv-Cre; Addgene) or pBabe-CreERT2-Hygro followed by selection in 200 μg/ml hygromycin (Sigma).

### Generation of human cells deficient in eIF2αP

Cells were infected with pMSCV retroviruses containing an HA-tagged form of human eIF2αS51A cDNA and the gene encoding for the green fluorescence protein (GFP) under the control of an internal ribosome entry site (IRES). After infection, GFP-positive cells were sorted out by flow cytometry and re-infected with either pGIPZ insert-less lentiviruses (control) or pGIPZ lentiviruses expressing an shRNA specific for the 3′ UTR of human eIF2α mRNA (Open Biosystems). Polyclonal populations were established by selection of cells in 2.5 μg/ml puromycin (Sigma).

### Senescence-associated β-galactosidase assay

Staining of cells and tumors for SA-β-Gal was performed as previously described [43,44].

### Cell cycle analysis

Cells were subjected to propidium iodide (PI) staining and FACScan analysis based on a previously described protocol [45]. FACS was performed with BD FACScalibur and the data was analyzed using the FlowJo software (Tree Star inc).

### ROS measurement and γ-H2AX staining

Endogenous ROS levels were quantified by incubating the cells with 5 μM 2′,7′-dichlorodihydrofluorescein diacetate (Molecular Probes) or 2.5 μM Cell-RoxTM Deep Red reagent (Molecular Probes) and FACscan analysis according to manufacturer's specifications. γ-H2AX staining was performed as described [46]. Cells were stained with 0.1 μg/ml 4′,6-diamidino-2-phenylindole (DAPI) for visualization of the nucleus.

### Colony formation assays

Cells (2×10^4^) were seeded and maintained in 6-well plates for 7 days. Visualization of colonies was performed by crystal violet staining as described [47]. Scanning of the plates was done with an Oxford Optronix Scanner and quantification was done by using Gel Count software.

### Western Blot analysis

Protein extraction and immunoblotting were performed as described [30]. The antibodies used were: rabbit monoclonal against phosphorylated eIF2α at S51 (Novus Biologicals), mouse monoclonal eIF2α (Cell Signaling), mouse monoclonal antibody against actin (Clone C4, ICN Biomedicals Inc), rabbit polyclonal against p53 (Novacastra), mouse monoclonal against Mdm2 [48], and mouse monoclonal against Rb or p21 (BD Biosciences). All antibodies were used at a final concentration of 0.1-1 μg/ml. Following incubation with the indicated primary antibodies and washes, membranes were probed with anti-mouse or anti-rabbit IgG antibodies conjugated to horseradish peroxidise (HRP) (Mandel Scientific). Proteins were visualized with the enhanced chemilumi-nescence (ECL) reagent (Thermo Scientific) detection system according to the manufacturer's instructions. Quantification of protein bands was performed by densitometry using Scion Image from the NIH.

### Xenograft tumor assays

Injection of cells in female athymic nude mice (Charles River Inc.) and tumor monitoring were performed as described [49]. Mice were treated with 4mg/kg doxorubicin delivered by intraperitoneal injections twice per week. The animal studies were performed in accordance with approved protocols and regulations by the Animal Welfare Committee of McGill University (protocol #5754).

### Statistical analysis

Error bars represent standard error as indicated and significance in differences between arrays of data tested was determined using two-tailed Student T test (Microsoft Excel).

## SUPPLEMENTARY FIGURES


