# Supplementary material for: eIF2α phosphorylation bypasses premature senescence caused by oxidative stress and pro-oxidant antitumor therapies
Source: Aging (Albany NY). 2013 Dec 9;5(12):884–901. doi: 10.18632/aging.100620 (PMC3883705; doi:10.18632/aging.100620)
Supplement: Supplementary file 1 [file aging-05-884-s001.pdf]

SUPPLEMENTARY FIGURES

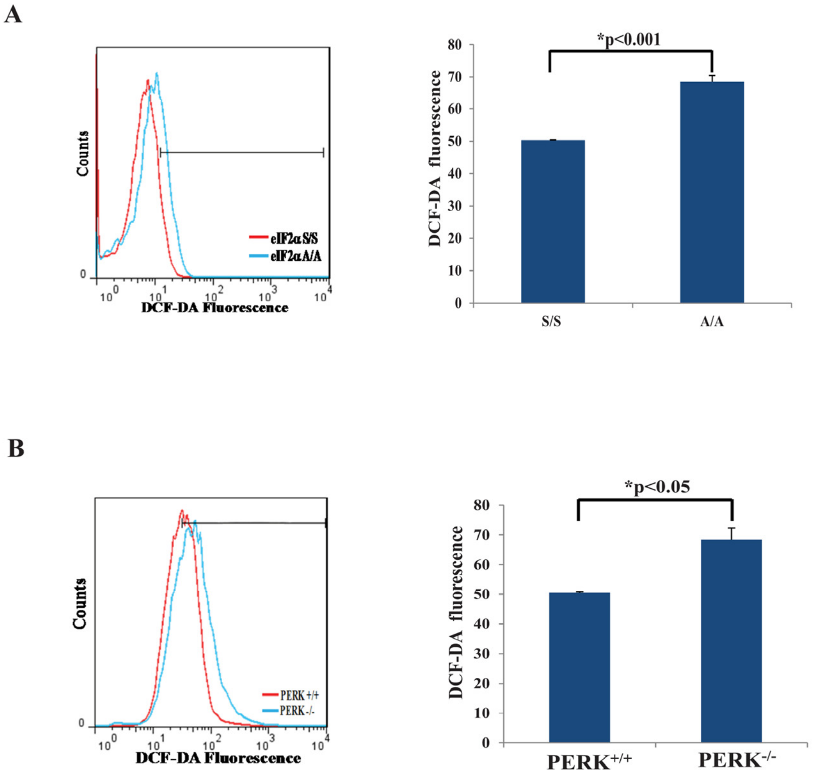

Suppl. Figure 1. Increased ROS production in eIF2αP- or PERK-deficient cells. Detection of endogenous ROS levels by staining with 2',7'-dichlorodihydro-fluorescein diacetate (DCF-DA) and FACS analysis in primary eIF2αS/S, eIF2αA/A MEFs (**A**) and in primary PERK$^{+/+}$ and PERK$^{-/-}$-MEFs (**B**) in passage 3. (**A, B**) Histograms represent the average ROS levels measured by DCF-DA fluorescence from three independent experiments.

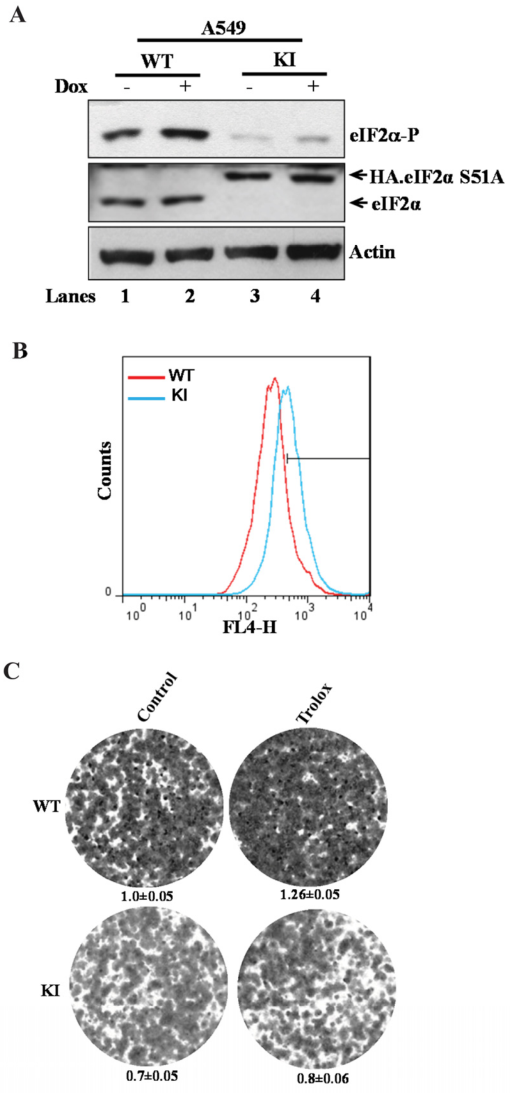

Suppl. Figure 2. Inactivation of eIF2αP in A549 cells decreases proliferation and increases. ROS. (A) Cell extracts (50 µg of protein) from wild-type (WT) and knock-in (KI) A549 cells treated with 30 nM doxorubicin (Dox) for 36 hrs were immunoblotted for the indicated proteins. Note the slower migration of the HA-eIF2αS51A in knock-in (KI) cells (lanes 3,4) compared to endogenous eIF2α in wild-type (WT) cells (lanes 1,2). (**B**) Production of ROS levels in A549 cells was assessed by Cell-RoxTM Deep Red staining and FACS analysis. (**C**) Colony formation assays of A549 WT and KI cells in the absence or presence of 200 µM Trolox. Cells were stained with crystal violet. Values represent optical density (OD) in arbitrary units.

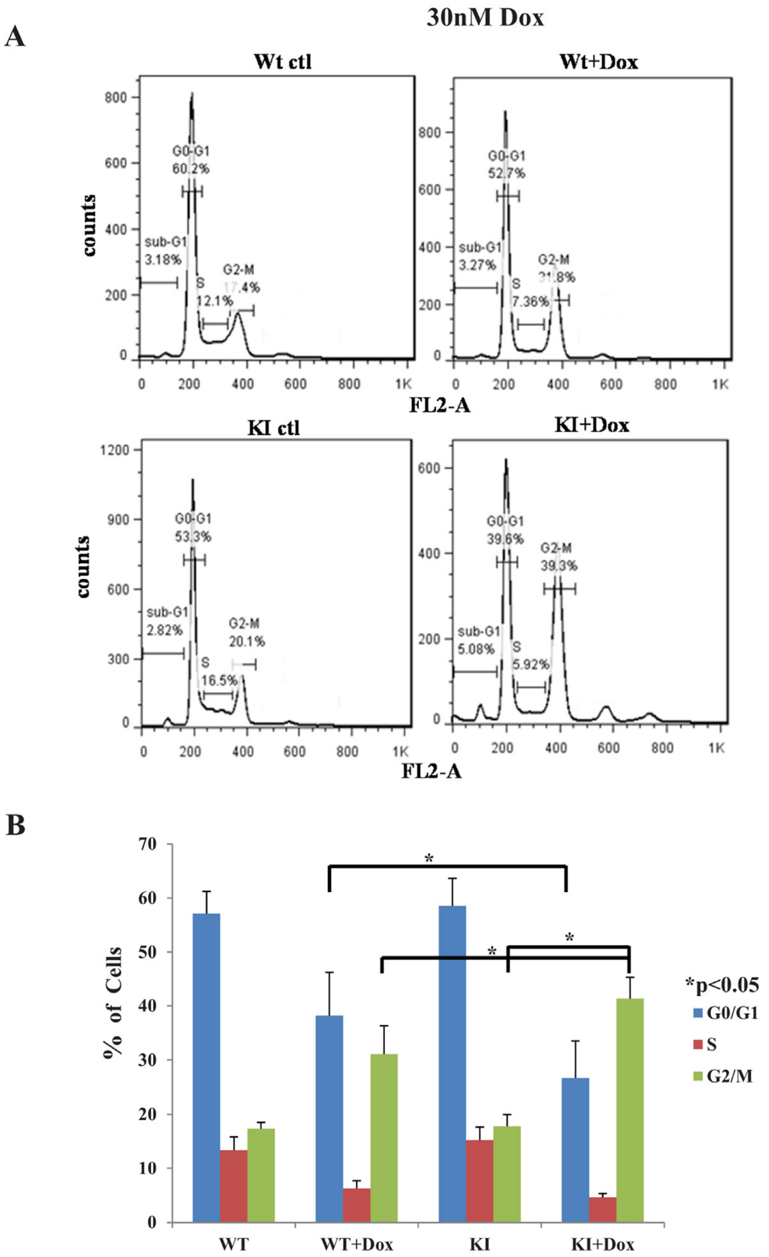

Suppl. Figure 3. Cell cycle analysis of tumor cells in response to treatment with low dose of doxorubicin. HT1080 WT and KI cells were left untreated or treated with 30 nM doxorubicin (Dox) for 36 hours. Cells were subjected to PI staining and FACS analysis. (**A**) Representative figure depicting the cell cycle distribution of cells treated with 30 nM of doxorubicin. **(B)** Histograms represent the average values of percentages of cells in G_0/G_1, S and G_2/M from three independent experiments.
